# The Use of Ovarian Vein Sampling to Lateralize a Virilizing Leydig Cell Ovarian Tumor

**DOI:** 10.1016/j.aace.2023.07.003

**Published:** 2023-07-22

**Authors:** Kirun Baweja, Shirley Shuster, Sara Awad

**Affiliations:** 1 Department of Medicine, Queen’s University, Kingston, Ontario, Canada; 2 Division of Endocrinology and Metabolism, University of Toronto, Toronto, Ontario, Canada; 3 Division of Endocrinology and Metabolism, Queen’s University, Kingston, Ontario, Canada

**Keywords:** hyperandrogenism, postmenopausal virilization, Leydig cell tumor, ovarian vein sampling

## Abstract

**Background/Objective:**

Leydig cell tumors are a rare androgen-secreting ovarian tumor. We present a patient with virilization symptoms secondary to a Leydig cell tumor, with nonrevealing imaging studies, that was localized using ovarian vein sampling (OVS).

**Case Report:**

A 56-year-old postmenopausal woman was referred by her gynecologist to the endocrinology clinic for voice-deepening, clitoral enlargement, scalp hair loss, and excessive body hair growth. Her total testosterone was 11.5 (0.3-1.3 nmol/L), bioavailable testosterone was 7.19 (0.1-0.6 nmol/L), and dehydroepiandrosterone sulfate was 4.0 (0.8-4.9 μmol/L). Transvaginal ultrasound and abdominal magnetic resonance imaging showed no adrenal or ovarian masses bilaterally. On adrenal vein sampling (AVS) and OVS, total testosterone from the left gonadal vein was 780.0 (0.3-1.3 nmol/L) and right gonadal vein was 18.6 (0.3-1.3 nmol/L), with a left-to-right ovarian testosterone ratio of 41.94. A bilateral salpingo-oophorectomy was performed, and a 1.0 cm Leydig cell tumor in the left ovary was noted on histopathology. One month after surgery, her total and bioavailable testosterone were <0.4 (0.3-1.3 nmol/L and 0.1-0.6 nmol/L, respectively). At 6 months, she had normalization of her voice to baseline, decreased clitoral size, decreased hair growth on her back, and improvement in her male-pattern baldness.

**Discussion:**

OVS and AVS are useful diagnostic investigation tools in cases of virilization, in which imaging is nonrevealing. Our case supports previously suggested left-to-right ovarian vein testosterone ratio of ≥15 being associated with a left-sided tumor.

**Conclusion:**

Few cases have been published on the interpretation of AVS and OVS in the setting of virilization. Previously suggested ratios for lateralization were valid for this patient.

**Highlights**

• This is a case of virilization in a post-menopause woman, with normal imaging results.
• Localization of the excess androgen production was achieved through adrenal vein sampling (AVS) and ovarian vein sampling (OVS).
• The patient underwent surgical removal and was found to have a Leydig cell ovarian tumour.
• There is no current consensus in interpretation of AVS and OVS in cases of virilization.
• This case supports previously suggested biochemical ratios for lateralization.

**Clinical Relevance**

We report our experience using adrenal vein sampling (AVS) and ovarian vein sampling (OVS) to lateralize the source of hyperandrogenism, causing virilization, in a postmenopausal woman. We discuss the specific use of AVS and OVS in cases of virilization in which an ovarian source is suspected but imaging is nonrevealing, as can often be the case with small androgen-secreting tumors. Our results support previously suggested ratios for localization using OVS.

## Introduction

Virilization is a clinical syndrome of rapidly progressive hirsutism, male-pattern baldness, deepening of the voice, and clitoromegaly, which should raise concern for an androgen-secreting ovarian or adrenal tumor. Ovarian tumors are a rare cause of postmenopausal virilization. The majority of ovarian tumors associated with hyperandrogenism are benign, commonly sex cord–stromal tumors^1^ and steroid cell tumors.^2^ This is in contrast to adrenal tumors, in which carcinomas are more likely to be androgenic than adenomas.^3^ Sertoli-Leydig cell tumors are a subtype of ovarian sex cord–stromal tumor and are especially rare, representing <0.5% of all ovarian neoplasms.^4^ These are often low-grade, and <20% of cases become clinically malignant.^5^^,^^6^

Ovarian vein sampling (OVS) and adrenal vein sampling (AVS) are important diagnostic tools in virilization cases in which an adrenal or ovarian source is suspected but imaging is nonrevealing. It involves cannulating the bilateral adrenal and ovarian veins to obtain blood for analysis of testosterone and dehydroepiandrosterone sulfate (DHEAS) levels. A peripheral vein blood sample is obtained for comparison. A consensus on interpretation of OVS and AVS results in this context has not been established, as there are few published cases of vein sampling for virilization and even fewer reporting results from all 4 veins because of low success rates of cannulation.^7^^,^^8^

We present a case of virilization in a 56-year-old woman who had nonrevealing imaging studies and underwent AVS and OVS to investigate and lateralize the source. She was found to have a left ovarian Leydig cell tumor on pathology after surgical removal.

## Case Report

A 56-year-old woman was referred to the endocrinology and metabolism clinic by her gynecologist for assessment of virilization. She had initially sought medical attention with her family physician for these symptoms and had been referred to a gynecologist for further investigation, who subsequently referred her to endocrinology. She had a past medical history of hypertension and prediabetes, and her medications were perindopril for blood pressure control and over-the-counter minoxidil drops for hair loss. She denied using any other over-the-counter or topical medications. Her symptoms began postmenopause at the age of 52 years. She reported rapidly progressive deepening of her voice; scalp hair loss; enlargement of her clitoris; and excessive dark and long hair growth on her face, back, chest, and extremities. She recalled that her hair growth had preceded her other symptoms by 6 years. She reported regular menstrual cycles until menopause, with no history of infertility. Her family history was negative for history of virilization or ovarian tumors, and she did not have constitutional symptoms of fever, weight loss, or night sweats. She denied use of exogenous testosterone.

**Fig. 1:**
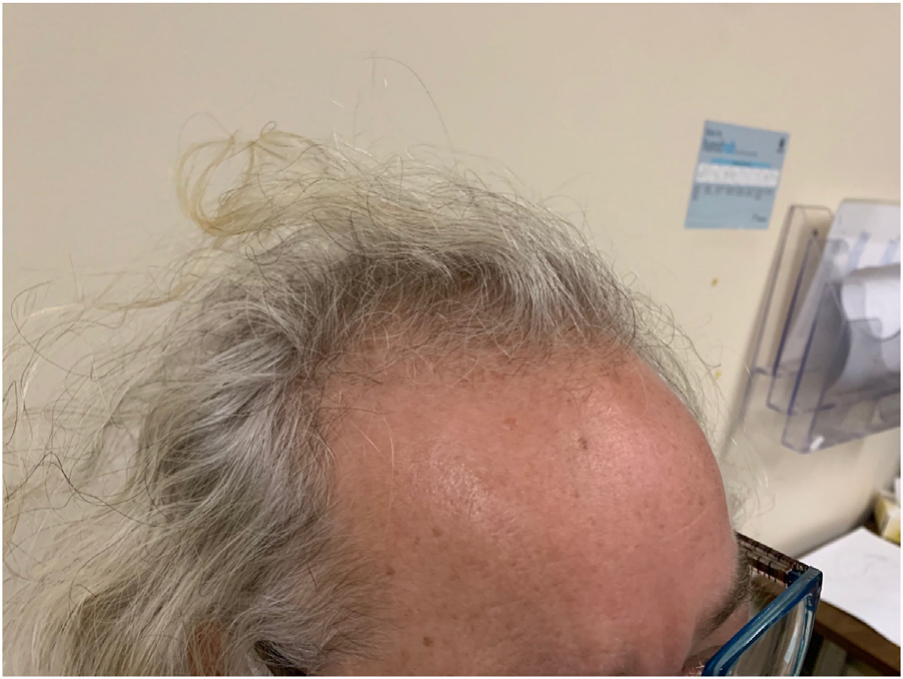
The patient’s male-pattern baldness in the vertex and temporal areas at the time of initial assessment in the endocrinology clinic.

Her physical examination was significant for deep voice; severe male-pattern baldness at the vertex and temporal areas (Fig.1); clitoris measuring >4 mm, consistent with clitoromegaly; and dark long hair growth around her chin, lower back, umbilicus, chest, and upper and lower extremities. Her Ferriman-Gallwey score was 20, representing moderate hirsutism.^9^ Her laboratory investigations showed total testosterone of 11.5 (0.3-1.3 nmol/L), with bioavailable testosterone of 7.19 (0.1-0.6 nmol/L). DHEAS was normal at 4.0 (0.8-4.9 μmol/L). Her other investigations, including thyroid-stimulating hormone, prolactin level, insulin-like growth factor 1, and growth hormone were within normal range (Table 1). Her cortisol suppressed to <50 nmol/L after 1 mg dexamethasone suppression test, and her early morning follicular phase 17-hydroxyprogesterone was 1.4 (0.4-1.5 nmol/L).

**Table 1:** Clinical Laboratory Results

Measure	Preoperative result	Postoperative result
Total testosterone (0.3-1.3 nmol/L)	**11.5** (↑)	<0.4
Bioavailable testosterone (0.1-0.6 nmol/L)	**7.19** (↑)	<0.4
Sex-binding hormone globulin (12-140 nmol/L)	18	
DHEAS (0.8-4.9 μmol/L)	4.0	3.3
TSH (0.40-4.50 mIU/L)	2.42	
Prolactin (5.0-27.0 μg/L)	4.9	
IGF-1 (48-235 μg/L)	175	
GH (<2.0 μg/L)	<0.1	
17-hydroxyprogesterone (0.4-1.5 nmol/L)	1.4	
Anti-Mullerian hormone (0.0-8.2 pmol/L)	<0.6	
Estradiol (<100 pmol/L)	86	

Abbreviations: DHEAS = Dehydroepiandrosterone sulfate; GH = growth hormone; IGF-1 = insulin-like growth factor 1; TSH = thyroid-stimulating hormone.

A pelvic transvaginal ultrasound showed 2 fundal uterine fibroids and normal appearance of the ovaries, with no abnormal adnexal masses. Magnetic resonance imaging of her abdomen and pelvis showed no adrenal lesions bilaterally, an anteverted uterus with 3 intramural fibroids, and no definite solid or cystic ovarian masses.

In the context of unremarkable biochemical and radiographic data, the patient underwent OVS and AVS. AVS showed right and left adrenal testosterone levels of 10.1 and 6.8 (0.3-1.3 nmol/L), respectively. OVS showed left ovarian testosterone level of 780.0 nmol/L (RR = 0.3-1.8) and right ovarian testosterone level of 18.6 nmol/L (RR = 0.3-1.3) (Table 2). Left-to-right ovarian testosterone ratio was 41.94.

**Table 2:** Ovarian and Adrenal Vein Sampling Results

Source	Total testosterone, nmol/L (RR = 0.3-1.3)	Androstenedione, nmol/L (RR = 0.7-2.6)	Cortisol (random) nmol/L (RR 65-470)	DHEAS, μmol/L (RR = 0.8-4.9)
Right gonadal vein	18.6	30.2	128	3.7
Left gonadal vein	780.0	97.7	114	3.6
Right adrenal vein	10.1	105.0	1425	5.5
Left adrenal vein	6.8	6.1	222	4.0
Peripheral vein	8.5	2.5	128	3.8

Abbreviations: DHEAS = Dehydroepiandrosterone sulfate; RR = risk ratio.

**Fig. 2:**
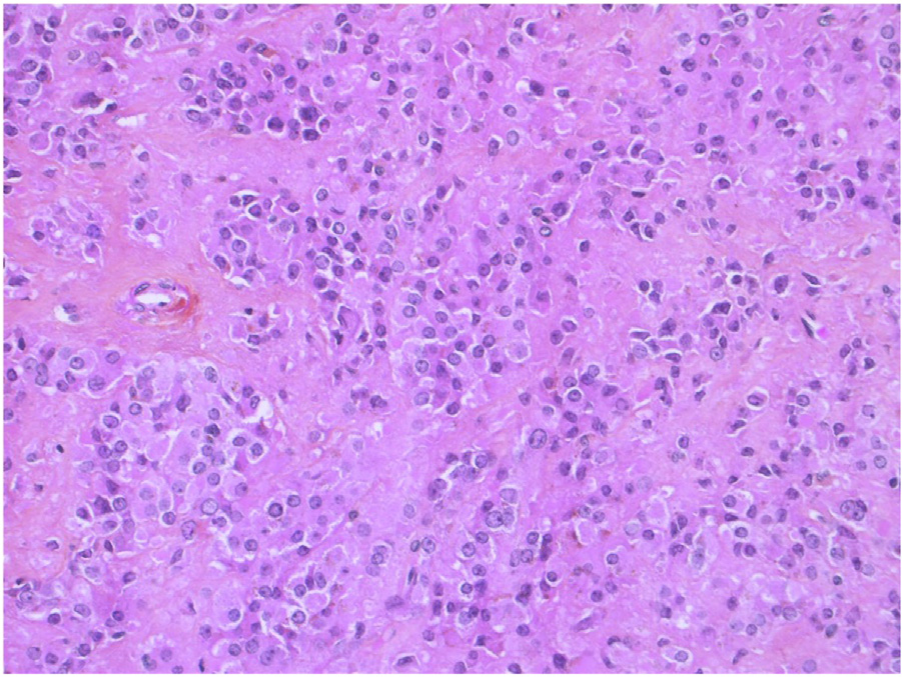
Left ovarian pathology after salpingo-oophorectomy, in keeping with Leydig cell ovarian tumor.

After discussion with gynecology-oncology at a multidisciplinary cancer conference, both left unilateral and bilateral salpingo-oophorectomy were offered to the patient. She opted for bilateral resection; therefore, bilateral salpingo-oophorectomy and total abdominal hysterectomy were performed. The histopathology showed a 1.0 cm left ovarian tumor composed of a monotonous cellular population that was positive for inhibin A, calretinin, and melan A (Fig. 2), most consistent with a Leydig cell tumor. The right ovary pathology was within normal limits. The uterine pathology showed leiomyomata in the myometrium and an inactive endometrium, with no evidence of hyperplasia, atypia, or malignancy.

One month after surgery, her total testosterone and bioavailable testosterone were both <0.4 nmol/L, and her virilization symptoms markedly improved at 6-month follow-up. She reported normalization of her voice to baseline, decrease in dark hair growth on her back, and improvement in her male-pattern baldness. She had moderate persistent hirsutism, requiring frequent facial hair removal and continued to use minoxidil for her baldness but felt that these had overall improved.

## Discussion

In this case, we present a patient with clinical signs and symptoms of virilization secondary to a Leydig cell tumor, a rare subtype of ovarian tumor that represents <0.5% of all ovarian neoplasms.^4^ Her initial laboratory investigations were notable for markedly elevated free and bioavailable testosterone, with a normal DHEAS level. This pattern suggested an ovarian source of androgen excess; in contrast, functional adrenal tumors generally secrete androgenic prohormones, resulting in elevated DHEAS.^10^ Her imaging revealed normal ovaries, with no adnexal masses. Given a high degree of clinical suspicion for an ovarian source of virilization, AVS and OVS were performed with the goal of lateralizing the source to allow for unilateral resection. Her left-to-right ovarian testosterone ratio was 41.94. Levens et al^11^ describe a left-to-right ratio ≥15 being strongly associated with a left-sided ovarian tumor; therefore, her ratio was felt to be in keeping with this. Her pathology revealed a Leydig cell tumor in the left ovary and normal right ovarian pathology, confirming our OVS result interpretation.

Most of ovarian stromal tumor cases are unilateral, without extraovarian spread, and fertility in premenopausal women is preserved through unilateral salpingo-oophorectomy. Bilateral oophorectomy is a reasonable option for postmenopausal patients and may be associated with improved survival rates, although current evidence is not definitive.^12^ Additionally, either endometrial curettage or total hysterectomy must be performed to rule out concomitant endometrial pathology. Although her OVS and AVS work-up would have allowed for unilateral resection, this patient opted for bilateral salpingo-oophrectomy with total abdominal hysterectomy as she was postmenopausal and was not interested in fertility preservation.

This case demonstrates the diagnostic challenge associated with such tumors, particularly given their difficulty to visualize radiologically. Patients should initially undergo both a transvaginal ultrasound study to visualize the ovaries and computed tomography or magnetic resonance imaging of the abdomen and pelvis to visualize the adrenal glands and ovaries. AVS and OVS should be considered as part of the subsequent work-up if imaging results are inconclusive and nonlocalizing or if only a small adrenal adenoma is identified, as localization of the culprit lesion is required for unilateral surgical resection.^13^ Although the low serum DHEAS in this patient favored an ovarian origin, sampling of both the ovarian and adrenal veins is still crucial, given reports of patients with androgen-secreting adrenal tumors and normal DHEAS levels.^7^

A consensus on interpretation of AVS and OVS results for hyperandrogenism has not yet been established. Moltz et al^14^ suggested that unilateral elevation of the ovarian-peripheral testosterone gradient >2.7 ng/mL warrants surgical exploration of the ovary. Levens et al^11^ found that in patients with elevated peripheral testosterone (≥4.5 nmol/L), a right-to-left ovarian testosterone ratio ≥1.44 correctly identified 90% of right-sided tumors, with a lower ratio suggestive of a left-sided lesion or bilateral lesions. A left-to-right ratio of ≥15 was strongly associated with a left-sided tumor.^11^ Our case supports these values.

Another noted abnormality on this patient’s vein sampling was her right adrenal androstenedione being unusually high compared to the left adrenal level. The left ovary was felt to be the predominant source, given the high testosterone ratio; therefore, bilateral salpingo-oophorectomy was pursued. Should the androgen levels have remained persistently elevated postresection, a right adrenal contribution would have been considered. Her androgen levels normalized after surgery, highlighting that mild noncausative AVS abnormalities can be present in cases of an ovarian source of hyperandrogenism.

The limitations of OVS and AVS are the lack of guidelines for interpretation in cases of virilization, given low procedural volume and low success rates in cannulating all 4 vessels. Reported success rates range from 27%^7^ to 45%^8^ for cannulation of bilateral ovarian and adrenal veins. The low rates may be because of anatomical variants as well as the presence of competent valves in ovarian veins of nulliparous women, the predominant demographic for these tumors.^7^ Owing to these challenges, venous sampling should only be performed after failure to identify a source on imaging in patients with clinical history suggestive of androgen-secreting tumor and at centers that are highly specialized in venous sampling procedures.

## Conclusion

OVS and AVS add diagnostic value in identification and localization of androgen-secreting tumors, particularly in cases when imaging is unrevealing. There is a lack of consensus on the interpretation of OVS results for lateralization because of the few cases currently published. Our case reports findings from all 4 veins and supports previously identified ratios. Guidelines may be established as more cases are reported and further studies emerge.

## Disclosure

The authors have no multiplicity of interest to disclose.
